# Transitioning peanut oral immunotherapy to clinical practice

**DOI:** 10.3389/falgy.2022.974250

**Published:** 2022-08-26

**Authors:** S. Lazizi, R. Labrosse, F. Graham

**Affiliations:** ^1^ Division of Allergy and Clinical Immunology, Department of Medicine, Centre Hospitalier de l’Université de Montréal, Montreal, QC, Canada; ^2^ Department of Pediatric Allergy and Immunology, Centre Hospitalier Universitaire Sainte-Justine, Montreal, QC, Canada

**Keywords:** oral immunotherapy (OIT), peanut allergy, food allergy, desensitization, sustained unresponsiveness, clinical tolerance induction, shared decision making

## Abstract

Peanut allergy is on the rise in industrialized countries, affecting 1%–4.5% of children and generally persisting into adulthood. It is associated with a risk of severe anaphylaxis and is one of the major causes of food allergy-induced deaths. Health-related quality of life is significantly impaired for patients and affected families due to food restrictions attributable to omnipresent precautionary allergen labeling, constant risk of potentially life-threatening reactions, and limitation of social activities. Oral immunotherapy (OIT) has emerged as a valid treatment option for patients with IgE-mediated peanut allergy, with randomized controlled trials and real-life studies showing a high rate of desensitization and a favorable safety profile, especially in young children. Ultimately, the decision to initiate peanut OIT relies on a multidisciplinary shared decision-making process, involving open, personalized and evidence-based discussions with patients and their caregivers.

## Background

Peanut allergy is on the rise in industrialized countries, affecting 1%–4.5% of children and generally persisting into adulthood (1–4). It is associated with a risk of severe anaphylaxis and is one of the major causes of food allergy-induced deaths (5). Health-related quality of life is significantly impaired for affected families (6) due to food restrictions attributable to omnipresent precautionary allergen labeling (PAL), constant risk of potentially life-threatening reactions, and limitation of social activities (7). Strict dietary avoidance and emergency treatment of accidental reactions with an epinephrine auto-injector is the current standard of care. Nonetheless, accidental reactions occur frequently with an annual incidence of 12%–14% (8, 9), leading many patients to seek alternative treatment options.

Oral immunotherapy (OIT) is increasingly recognized as a valid option for the treatment of IgE-mediated food allergy, with many studies focusing on peanut OIT. It involves daily ingestion of a food allergen with the goal of increasing allergen tolerance (10). OIT protocols usually consist of 3 steps: initial dose escalation, up-dosing or buildup phase and maintenance phase (11). The initial dose escalation determines the starting dose, which is followed by dose increases every 1–2 weeks over several months until a predefined maintenance dose is reached. This maintenance dose is then ingested every day for a prolonged period. This continuous exposure is expected to modulate the immune response against the allergen, resulting in a decreased production of IgE and increased production of neutralizing IgG4 antibodies (10).

In this review, clinical desensitization is defined as a state in which a patient can tolerate peanuts as long as regular ingestion occurs. Sustained unresponsiveness is defined as a state in which the patient who is desensitized can stop eating the food for a short period of time (generally 4–8 weeks but up to 6 months in some studies) and remains non-reactive when the food is reingested. Oral tolerance refers to permanent resolution of peanut allergy, a state where tolerance is maintained even after prolonged period of peanut avoidance (i.e., patient can eat peanuts at any given dose and time without regular ingestion). The purpose of this review is to examine data on peanut OIT efficacy and safety and to discuss evidence towards transitioning peanut OIT into clinical practice.

## 1. Efficacy of peanut OIT

Several randomized controlled trials (RCTs), uncontrolled studies, and clinical practice retrospective chart reviews have demonstrated the efficacy of peanut OIT at inducing clinical desensitization to peanuts (Table 1). Generally, clinical desensitization rates vary between 60 and 80% in clinical trials, and 80%–90% in outpatient practice. PALISADE is the largest peanut OIT RCT (using a peanut-derived drug containing up to 300 mg of protein) and was conducted by Vickery et al. in 2018 (27). The primary endpoint was tolerance of 600 mg peanut protein on an exit double-blind, placebo-controlled food challenge (DBPCFC). Of 496 children aged 4–17 years old, 67.2% were able to tolerate the dose of 600 mg compared to 4% in the placebo group. ARTEMIS is another recent peanut OIT RCT using the same peanut-derived drug, in which 58% of 132 children aged 4–17 years old tolerated 1,000 mg of peanut protein on an exit DBPCFC after 3 month maintenance at 300 mg of peanut protein (34). Afinogenova et al. described the largest retrospective study of patients having undergone peanut OIT. A chart review of 783 patients aged 3.5–48.3 years old who underwent peanut OIT at the New England Food allergy treatment center showed a desensitization rate of 89% (33). All in all, studies point to a high desensitization rate with peanut oral immunotherapy in children, although more data is needed in adults (10). One important caveat is that desensitization may not be the most adequate efficacy endpoint, and reactions in the community may be a better surrogate marker of clinical efficacy (38, 39), which is an important point to be addressed in future studies.

**Table 1:** Peanut OIT studies and efficacy outcomes.

Studies	Design	*N* ^a^	Age (years) Median [Range]	Peanut sIgE Median	Maintenance Dose^b^	DSS (ITT)	SU (ITT)
Clark 2009 (12)	Open	4	12.5 [9–13]	55.6 (Mean)^c^	800	100%	NA
Jones 2009 (13)	Open	39	4.79 [1–9.25]	85.4	300–1,800	69%	NA
Blumchen 2010 (14)	Open	23	5.6 [3.2–14.3]	95.6	125	61%	13%
Varshney 2011 (15)	RCT	19	7 [3.16–10.5]	106	Up to 4,000	84%	NA
Anagnostou 2011 (16)	Open	22	11 [4–18]	29.7	800	64%	NA
Anagnostou 2014 (STOP II) (17)	RCT	49	12.4 [7–16]	NS	800	49%	NA
Wasserman 2014 (18)	R	352	[3–24]	NS	415–8,000	85%	NA
Vickery 2014 (19)	Open	24	[1–16]	NS	1,800–4,000	NA	31%
Syed 2014 (20)	RCT	23	10.4 [5–45]	100	Up to 4,000	87%	30%/13%
Narisety 2015 (21)	RCT^d^	11	11.1 [9.7–13]	169	2,000	64%	36%
Vickery 2017 (DEVIL) (22)	Open (LD/HD)	20/17	2.4 [0.75–3]	14.4	300/3,000	85%/76%	85%/71%
Kukkonen 2017 (23)	RCT	39	8.3 [6.3–18.6]	10	800	67%	NA
Bird 2018 (24)	RCT	29	7 [4–21]	64.3	300 (AR101)	79%	NA
Fauquert 2018 (PITA) (25)	RCT	21	14.5 (Mean)	162 (Mean)	400	81%	NA
Nagakura 2018 (26)	Open	24	9.6 [6.1–16.2]	55.4	133	58.3%	33.3%
Vickery 2018 (PALISADE) (27)	RCT	372	[4–55]	69	300 (AR101)	67.2%	NA
Reier-Nilsen 2019 (TAKE-AWAY) (28)	RCT	57	10.1 [5.2–15.2]	52 (Mean)	Up to 5,000	61%	NA
Blumchen 2019 (29)	RCT	31	6.6 [4.8–9.8]^e^	89.5	Up to 250	74.2%	NA
Wasserman 2019 (30)	R	270	8.1 (Mean) [4–18]	24.1	2,000	79%	57.9%
Soller 2019 (31)	R	270	1.9 [1.25–2.75]^e^	5.03	300	90%	NA
Chinthrajah 2019 (32)	RCT	85	10 [9–13]	75.7	4,000	85%	13% (52w)
Afinogenova 2020 (33)	R	783	9.7 [3.5–48.3]	53.1 (Mean)	625–3,750	89%	NA
O’B Hourihane 2020 (ARTEMIS) (34)	RCT	132	9 [4–17]	43.5	300 (AR101)	58%	NA
Jones 2022 (IMPACT) (35)	RCT	146	3.3 [0.6–3.7]^e^	54.6	2,000	71%	21%
Yahia 2022 (36)	Cross-sectional	28	3.4 (Mean)	6,5 (Mean)	300	82.1%	NA
Chu 2022 (PISCES) (37)	RCT	17/16	8.1/7.8 (Mean)	79.81/76.17 (Mean)	500^f^/500	53%/75%	NA

DD, Daily dosing; DSS, Desensitization; HD, High dose; ITT, Intention to treat; IQR, Interquartile range; LD, Low dose; NA, Not applicable; NDD, Non-daily dosing; NS, Not specified; R, Retrospective; RCT, Randomized controlled trial; sIgE, Specific Immunoglobulin E; SLIT, Sublingual immunotherapy; SU, Sustained unresponsiveness.

^a^ in treatment group.

^b^ mg of peanut protein.

^c^ extrapolated from Table 1 of (12).

^d^ OIT vs SLIT.

^e^ IQR.

^f^ Peanut OIT + Anti-H1/H2.

## 2. Peanut OIT adverse reactions

The intention-to-treat desensitization rates presented above (Table 1) show that approximately 15%–20% of study participants fail to reach desensitization targets by the end of the study period. The reasons for this are numerous and include treatment discontinuation due to OIT-related side effects, non-compliance, relocation, or uncontrolled asthma among others.

Adverse reactions during OIT are reported in 50%–95% of patients (27, 40), depending on the severity of the peanut allergy. The most frequently reported symptoms are generally mild, and usually include itching of the mouth and throat and abdominal discomfort. Between 10% and 25% of patients experience severe allergic reactions requiring the injection of epinephrine when taking doses at home (18, 22, 33, 40). One study reports a rate of 9% of patients requiring at least one dose of epinephrine during physician supervised up-dosings (35).

Cofactors are external factors that can influence the reactivity threshold of patients undergoing peanut OIT and are sometimes implicated in allergic reactions at home to doses that were previously well tolerated. These include physical exertion, infections, extreme heat, poorly controlled asthma, alcohol consumption, use of non-steroidal anti-inflammatory drugs (e.g., ibuprofen), viral infections, sleep deprivation, as well as menstrual cycles (41). When beginning OIT, patients are given precise instructions to minimize the impact of these cofactors surrounding daily doses. For example, they are instructed to reduce their doses to half during viral illnesses, and to avoid exercise 1 h before and 2 h after their doses (42). These recommendations generally stay in effect even at the maintenance stage.

In addition to dose-related immediate reactions, eosinophilic esophagitis (EoE) has been associated with OIT in up to 2.7% of cases (43), although this seems to be a temporary effect that usually resolves upon discontinuation of treatment (44). This is distinct from spontaneous EoE, developing in the absence of OIT, which usually has a chronic clinical course. Interestingly, esophageal eosinophilia was seen during dose escalations in the majority of patients undergoing peanut OIT but resolved without treatment during maintenance phase (45). Other GI manifestations include eosinophilic esophagitis like oral immunotherapy related syndrome (ELORS), which manifests as delayed vomiting usually around 2–6 h after doses and is accompanied by peripheral eosinophilia (46, 47). Lastly, peanut aversion can develop in a substantial portion of patients and can limit treatment compliance, and lead to treatment failure in some cases (42, 48). Importance is placed on varying textures and food formats to improve palatability of OIT peanut doses (see Table 2 for peanut maintenance dose food equivalences), especially in children.

**Table 2:** Peanut maintenance dose examples with food equivalents.

Food	Peanut	Peanut butter	Peanut flour	Reese's pieces (brand)^a^	Peanut M&M's (brand)^b^	Bamba (brand)^c^
Peanut protein concentration	≈250 mg/peanut	3 g/15 ml^d^	6 g/30 ml^e^	4 g/40 g ≈75 mg/piece	3 g/12 pieces^f^ ≈250 mg/piece	≈80 mg/stick^g^
250 mg equivalent	1	¼ teaspoon	¼ teaspoon	3	1	3
1,000 mg equivalent	4	1 teaspoon	1 teaspoon	13	4	12
3,000 mg equivalent	12	3 teaspoons	3 teaspoons	40	12	37

**NB, It is always advisable to adapt calculations based on protein content specified on labels as local variations may exist.**

^a^ Reese's pieces: The Hershey Company, Hershey, PA.

^b^ M&Ms: Mars Chocolate, Hackettstown, NJ.

^c^ Bamba: Osem Food Industries, Shohan, Israel.

^d^ Varies between 3 and 4 g/15 ml depending on brands. Best to use smooth peanut butter to avoid large peanut chunks.

^e^ Varies between 5 and 6 g/30 ml depending on brands.

^f^ Some pieces may be peanut-free.

^g^ From (49).

## 3. Potential benefits of peanut OIT

### 3.1. Protection against accidental reactions

The increase of reactivity threshold achieved by OIT leads to a reduction of accidental reactions to peanuts. Baumert et al. performed a quantitative assessment of risk reduction following OIT and found that increasing the baseline threshold from 100 mg or less to 300 mg of peanut protein post-OIT would reduce the risk of reacting to foods with precautionary allergen labeling by more than 95% (50). Further increasing the threshold to 1,000 mg would provide an additional risk reduction of 70-fold. This has also been suggested by a clinical trial where peanut OIT was found to reduce the number and severity of allergic reactions after accidental peanut consumption compared to placebo (51).

### 3.2. Improvement of quality of life

Open trials with peanut OIT have described a significant improvement in quality of life (QoL) in patients undergoing the treatment. Several studies have shown a statistically and clinically significant effect on the QoL with OIT using standardized questionnaires such as the Food Allergy Quality of Life Questionnaire (FAQLQ). More specifically, there was an improvement in emotional impact and a decrease in hypervigilance, social limitations, dietary restrictions and anxiety regarding diet (17, 52–56). QoL tends to improve significantly upon reaching the maintenance phase but may be preceded by a temporary detrimental effect on QoL during up-dosing (57).

## 4. Limitations of peanut OIT

### 4.1. Sustained unresponsiveness

While data has clearly demonstrated the effectiveness of peanut OIT in inducing desensitization in a large portion of patients, data concerning sustained unresponsiveness is limited and, although variable, rates are generally under 50% (Table 1). This depends on numerous factors including age, length of avoidance and duration of the maintenance phase. Blumchen et al. reported a sustained unresponsiveness rate of barely 13%, after a short treatment of 9 months, with a therapeutic target set at 500 mg of protein (approximately 2 peanuts) (14). Vickery et al, on the other hand, reported a 50% sustained unresponsiveness rate with a higher maintenance dose and longer protocol duration (median of 5-year treatment with a maintenance dose of 4,000 mg peanut protein) (19). Interestingly, Chinthrajah et al. performed up-dosings up to a maintenance dose of 4,000 mg of peanut protein for nearly 2 years and patients were then split in two groups for 52 weeks: discontinuation or dose lowering to 300 mg of peanut protein (32). Sustained unresponsiveness in the group discontinuing treatment gradually decreased throughout the study period reaching a low of 13% (8/60) after 52 weeks. This study highlights the fact that sustained unresponsiveness decreases with increasing duration of peanut avoidance (32).

Thus, patients beginning treatment should be warned that long-term (lifetime) ingestion of peanuts may be necessary to maintain desensitization. One important aspect to mention is that in patients presenting a return of clinical reactivity after a period of avoidance (failed sustained unresponsiveness), the threshold of reactivity to the allergen is generally still higher than before OIT.

Adjuvanted peanut OIT studies are ongoing with the objective to improve long-term sustained unresponsiveness and/or increase the safety profile of OIT (58). Examples of adjuvants currently being studied in peanut OIT trials include omalizumab (59), dupilumab (NCT03682770), abatacept (NCT04872218), prebiotics (ACTRN12617000914369) and probiotics. Tang et al. notably reported a very high sustained unresponsiveness rate of 82% with a peanut OIT protocol combined with probiotic Lactobacillus rhamnosus in children after a short 2-week discontinuation period (60). Unfortunately, the clinical trial did not include a probiotic-free OIT arm and the 2-week period was relatively short compared to other studies, which may have led to an overestimation of the sustained unresponsiveness rate. Indeed, a more recent phase 2b trial by the same group found a sustained unresponsiveness rate of 46% in the peanut probiotic group compared to 51% in the peanut OIT without probiotic group (not significantly different) (61). Future studies will hopefully provide a better understanding of the pathomechanisms associated with sustained unresponsiveness and tolerance in oral immunotherapy to help develop more effective adjuvants.

### 4.2. Side effects compared to avoidance

A recent meta-analysis showed that peanut OIT is associated with higher risk of anaphylactic reactions requiring epinephrine than avoidance (62). Anaphylaxis can occur both during escalation period and maintenance doses. It is important to note that these reactions occur in a controlled environment (at home) and are an expected potential side effect of treatment, discussed prior to obtaining informed consent from the patient, as opposed to accidental reactions in the community, which generally occur in unprepared social situations (63). OIT is expected to decrease long-term and accidental reactions (51, 63), which could justify having potential increased reactions during up-dosing and beginning of maintenance phase. Furthermore, studies that were excluded from PACE include real-life observational data which have shown a high safety profile for peanut OIT (22, 31). All in all, the risk of anaphylaxis during OIT should always be discussed before providing OIT, and patients should have a personalized action plan to guide them in the acute management of anaphylaxis.

### 4.3. Quality of life

Although longer-term quality of life seems to improve (see previous section), OIT comes with a lot of constraints, especially related to cofactor avoidance during treatment. This can lower the quality of life in certain patients (64). OIT is also time-consuming and requires frequent clinic visits, which can lead to absenteeism from work and school.

Thus, prior to initiation, OIT risks and benefits should be discussed with a focus on the patient and their family's objectives to allow shared decision-making (65).

## 5. Transitioning peanut OIT to clinical practice

### 5.1. Data from community practice OIT

A growing number of allergists in North America have started adopting OIT in private practice. In a survey, 13.8% of allergists said they had already incorporated OIT in their practice (66). In clinical practice, OIT has generally been effective with clinical desensitization rates of 80%–90% (18, 30, 31, 33). Wasserman et al. recently published clinical experience with 270 patients undergoing peanut OIT in private practice (30). Dose escalations were achieved in 79% of patients. Adverse effects included grade I–II reactions in 170 patients, and grade III–IV reactions in 91 patients, whereas 63/270 (23%) incurred anaphylactic reactions requiring epinephrine (Mueller grading system (67)). The largest private practice peanut OIT study by Afinogenova et al. involving 783 patients showed a desensitization rate of 89% with 10% of patients experiencing systemic reactions during buildup and 19% during maintenance (33). These data support the successful transition of peanut OIT into clinical practice, with data from private practice showing high efficacy with safety parameters comparable to research protocols.

### 5.2. Patient selection

#### 5.2.1. Selecting the right patient

Selecting the right patients for OIT is crucial for treatment success. Factors associated with increased side effects of therapy include older age, high baseline specific IgE (sIgE), history of severe anaphylaxis, although these are not absolute contraindications to initiating therapy after risk-benefit discussion. However, one might refer these more challenging cases to tertiary specialized centers, whereas milder allergies could be treated in outpatient settings. Absolute contraindications for OIT initiation include uncontrolled asthma and pregnancy (10). Relative contraindications include active EoE, severe active atopic dermatitis, and relative contraindications to epinephrine (e.g., active heart disease); decision to initiate OIT in such situations should be based on clinical judgment and shared decision-making. In addition, patients who are unable to comprehend desensitization protocols, unmitigated language barriers, non-collaborative family dynamics, lack of commitment from patients or caregivers and non-adherence to protocol also constitute contraindications to peanut OIT (10).

#### 5.2.2. Should Peanut OIT Focus on Younger Children?

Data from the literature shows higher desensitization and sustained unresponsiveness rates as well as less frequent severe adverse events in toddlers when compared to older children, possibly due to greater immune plasticity (22, 31, 35). The DEVIL study showed that a maintenance dose of 300 mg or 3,000 mg is safe and effective in preschool children, achieving desensitization rates of 85% and 76% respectively (22). Sustained unresponsiveness after 4 weeks of avoidance was very high at 85% for the low dose and 71% for the high dose.

In a multicentric Canadian study, Soller et al. examined real-world safety of peanut OIT in 270 preschoolers who achieved a 90% desensitization rate (31). Grade I-II reactions occurred in 67.4% of participants, grade III-IV occurred in 0.4% of participants and epinephrine was required in only 4.1% of patients (World Allergy Organization Subcutaneous Immunotherapy Reaction Grading System (68)). A recent RCT by Jones et al. studying peanut OIT in children under 4 years old showed that 71% of 96 children achieved desensitization whereas only 21% achieved sustained unresponsiveness (Intention-to-treat) after 6 months of avoidance, which is a longer avoidance period than in most protocols (usually 4–8 weeks) (35). Nevertheless, younger patients had significantly higher rates of remission with 71% of children under 2 years of age achieving sustained unresponsiveness. Overall, remission was associated with younger age and lower baseline peanut-specific IgE (35). Thus, future peanut OIT resources may need to be invested in younger children to maximize positive long-term outcomes.

### 5.3. Practical aspects

#### 5.3.1. Peanut format

A major obstacle to widespread implementation of OIT is the lack of standardized product and the tedious process of preparing precise peanut doses. Pre-weighted doses of peanut powder such as PB2 peanut flour have been used in different clinical trials. Generally, these doses are weighed using a microbalance and individual doses are delivered to the patient in powder forms (e.g., in plastic cups). Doses are ingested daily by patients by mixing powders with soft foods such as apple sauce. Although effective, this process is time consuming and requires appropriate resources. Another option is to prepare a peanut suspension in simple syrup using peanut flour (50 mg/ml of peanut protein) (10). This was shown to add a gain in efficiency of 2,340% when compared to weighing powders (69) and may be an option to facilitate implementation of OIT in clinical practice. Once the patient reaches higher doses, switching powders or suspensions to readily accessible prepackaged foods or whole peanuts may be an easier option and less resource-intensive (see Table 2 **for maintenance dose equivalences**).

In the United States, the FDA recently approved Palforzia (AR101), a standardized good manufacturing practice (GMP) manufactured peanut powder derived from roasted peanut flour and premeasured in capsules with different dosages. The product is intended to simplify the preparation steps of peanut doses and provides a precise dose of peanut to the consumer undergoing OIT. Unfortunately, Palforzia costs $9,840 USD per year ($820 per month) and this high cost (100 times more than market-available peanut powder) could negatively affect widespread consumer access to this treatment. The product likely has no efficacy or safety advantage compared to shelf-bought peanut products, considering that slight peanut dose variations have not been shown to increase risk of dose-related reactions (70).

#### 5.3.2. Protocol

Another challenge in routine implementation of peanut OIT is the lack of a standardized protocols. This is partly because OIT should be personalized, and one cannot use a one-size-fits-all approach (10). As with all foods, peanut OIT involves an initial dose escalation, followed by up-dosing and maintenance phases (71). An initial dose escalation typically involves a fixed series of very low doses to identify the most sensitive patients (e.g., 0.1–6 mg of protein (22)); the patient then continues daily ingestion of the highest tolerated dose or the final dose if no reaction occurs. Peanut up-dosings are generally performed every 1 to 2 weeks in research protocols, although up-dosings can occur at longer intervals (up to 3 months (71)) depending on OIT provider resources and availabilities. After achieving the maintenance dose, the patient pursues daily dose ingestion (See Table 2 for peanut food equivalents for maintenance dose**)** and is generally re-assessed every 6–12 months, with discussion of adverse reactions and treatment compliance since last follow-up. Reduction in maintenance dosing frequency may sometimes be an option to counter long-term dosing fatigue (e.g., every other day, 3 times per week, or weekday dosing with weekends off), although there is a lack of data for optimal guidance. This is sometimes based on a successful high threshold OFC in clinical practice. In some situations, patients can be challenged for sustained unresponsiveness after a certain period of time has elapsed, based on clinical and immunological factors (e.g., fall in specific IgE levels, negativation of skin prick test) (30). More in-depth information about different protocols and maintenance doses can be found elsewhere in the literature and is beyond the scope of this review (10, 49, 71, 72).

#### 5.3.3. Resources and infrastructure

Another challenge of implementing peanut OIT in clinical practice is the reorganization of infrastructure. All physicians administering peanut OIT should be able to recognize and treat OIT-related symptoms and have access to the appropriate infrastructure and equipment to treat anaphylactic reactions. This includes a space to receive the patients, a designated room to prepare doses, an emergency treatment space and access to a service corridor to transport patients in case of reaction (10, 49). Moreover, the ability to safely and efficiently provide food challenges is an essential part of an OIT practice and not all allergists offer them because of perceived barriers such as time, space, staffing, and proximity to a hospital (73).

#### 5.3.4. Patient safety

Before initiating OIT, patients should have a personalized action plan to guide them in the management of adverse effects. Families should be equipped with an algorithm providing guidance for situations such as missed doses or illness (49). They should also be able to contact a professional to whom they can address questions and concerns during treatment. Some physicians offer 24/7 on call availability to their OIT patients (74), which can require significant personal commitment and time investment, especially in OIT practices with a large number of patients. Another more practical option is to offer OIT-related assistance only during work hours; outside of those hours, patients are instructed to follow their emergency care plan in case of dose-related reactions, which includes treating themselves with epinephrine and calling 911 in case of anaphylaxis (patients are always instructed to contact the clinic in case of anaphylactic reactions to their doses).

In the build-up phase, patients are generally monitored for 1 h after up-dosings (49), but the timeframe can vary depending on the clinical practice and increased surveillance could be necessary in higher-risk patients (e.g. patient with previous delayed reaction to up-dosing). In addition, physicians should be aware of the possibility of EoE and inquire about suggestive symptoms during follow-ups.

#### 5.3.5. Multidisciplinary approach and expertise

A multidisciplinary approach can be implemented to relieve the burden of certain tasks from OIT providers (10, 49). For instance, nurses can be an essential point of contact with patients and answer questions regarding doses and treatment of allergic reactions. They can additionally coordinate care with treating physicians. Dieticians can provide important information for food equivalents once maintenance dose is attained. Psychologists may be beneficial in patients with anxiety or stress related to their food allergy. Patient and food allergy support groups can provide an important outlet for discussing information that may not be readily available.

## Conclusions

With the advent of personalized medicine, OIT represents a novel therapeutic modality for IgE-mediated peanut allergy, wherein the status quo has been avoidance. A patient-centered discussion before initiating OIT might entail multiple visits to ensure adequate comprehension of benefits as well as limitations of this therapy. Although transition to clinical practice has already begun in many centers, obstacles to wider implementation include substantial infrastructure and human resource requirements. Further data is needed to better establish long-term outcomes such as sustained unresponsiveness. Younger patients (preschool children) seem to have more favorable long-term outcomes and should increasingly be prioritized for this form of treatment. Ultimately, the decision to initiate peanut OIT relies on a multidisciplinary shared decision-making process, involving open, personalized, and evidence-based discussions with patients and their caregivers.
